# Combined drug therapeutic strategies for the effective treatment of Triple Negative Breast Cancer

**DOI:** 10.1042/BSR20171357

**Published:** 2018-01-30

**Authors:** Naveen K.R. Chalakur-Ramireddy, Suresh B. Pakala

**Affiliations:** Biology Division, Indian Institute of Science Education and Research (IISER) Tirupati, Andhra Pradesh, India

**Keywords:** Cell Signalling Pathways, Drug Therapy, Triple Negative Breast Cancer

## Abstract

TNBC (Triple Negative Breast Cancer) is a subtype of breast cancer with an aggressive phenotype which shows high metastatic capability and poor prognosis. Owing to its intrinsic properties like heterogeneity, lack of hormonal receptors and aggressive phenotype leave chemotherapy as a mainstay for the treatment of TNBC. Various studies have demonstrated that chemotherapy alone or therapeutic drugs targeting TNBC pathways, epigenetic mechanisms and immunotherapy alone have not shown significant improvement in TNBC patients. On the other hand, a combination of therapeutic drugs or addition of chemotherapy with therapeutic drugs has shown substantial improvement in results and proven to be an effective strategy for TNBC treatment. This review sheds light on effective combinational drug strategies and current clinical trial status of various combinatorial drugs for the treatment of TNBC.

## Introduction

A search of term ‘triple-negative breast cancer’ in PubMed hits more than 7000 publications; of which 5000 were published in the last 5 years. TNBC (triple negative breast cancer) is an intrinsically heterogeneous disease which accounts for nearly 15–20% cases among 1.7 million new breast cancer cases diagnosed annually across the world [1].

Chemotherapy remains the mainstay for the treatment of TNBC due to lack of targeted therapies. Hormone-targeted drugs like tamoxifen, aromatase inhibitors and Her2-targeted drugs like trastuzumab are ineffective towards the treatment of TNBC due to the absence of receptors. A localized breast cancer can be primarily treated by surgery, while the metastasized breast cancer treatment focuses on improving the quality of life (QOL) by increasing the outcome of pCR (pathological clinical response), PFS (progression-free survival) and prolonging the OS (overall survival) rate of the patient. The rapidly increasing evidence of research and lack of therapeutic options show the significance of investigating effective therapeutic strategies for the treatment of TNBC.

## Molecular characteristics of TNBC

TNBC is a breast cancer subtype defined as lack of expression of hormonal receptors (oestrogen (ER) negative (<1%), progesterone (PR) negative (<1%) and HER2/neu) [2,3]. TNBC is a breast cancer subtype with similar characteristics of basal-like with an aggressive phenotype and high metastatic rate. TNBC exhibit properties of high histological grade [4] with distinct pathological and clinical features and associated with poor prognosis [5]. The 5-year survival rate for TNBC is 70% less than other breast cancer subtypes having 80% survival rates [6].

‘BRCAness’ can be defined as inherited and acquired mutations in DNA repair mechanisms in breast cancer cells [7]. BRCAness enriched phenotype in TNBC can be used as a biomarker for the exploitation of therapeutic options and clinical implications [8,9]. TNBC showed a high prevalence of BRCA mutations when compared with other subtypes of breast cancer [10–12]. Studies showed that 15–20% of TNBC patients carry BRCA1/2 germline mutations [10]. In recent years, gene expression signatures have been linked with TNBC to unravel distinct molecular subtypes [13]. TNBCs overlap up to 70% with basal-like breast cancer but are clinically and histopathologically distinct [14]. Based on the gene expression profiling and meta-analysis of 21 datasets of breast cancer, TNBCs are categorized into seven subclasses: Basal-like subclass (Basal-like 1 and Basal-like 2), Mesenchymal (M), MSL (mesenchymal stem-like), IM (immunomodulatory), LAR (luminal androgen receptor) and others. Identification of distinct TNBC subtypes may provide biomarkers for selection of patients in designing clinical trials and may help in the prediction of response to the treatment [13].

A study in 2006 showed that TNBC is linked to ethnic and menopausal differences which are not observed in ER+/Her2− and ER+/Her2+ breast cancer. The study also reports that prevalence of TNBC in African American women is 47%, twice when compared with white women which accounts only 22%, and this rate further increases to three-fold when considering factors like age and stage of diagnosis. African American premenopausal women diagnosed with breast cancer showed 39% of TNBC [15].

## Pathways and therapeutic targets in TNBC

**Figure 1:**
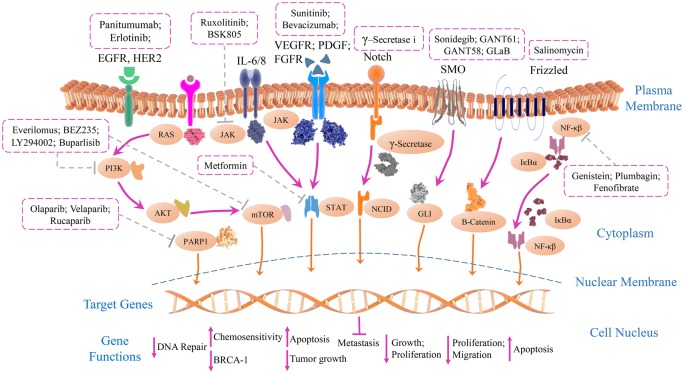
Overview of signalling pathways involved with identified potential inhibitors in TNBC The network of multiple signalling cascades with downstream effectors help in the maintenance of growth, proliferation, survival and metastasis of TNBC cells. The signalling pathways like NF-κB, PI3/AKT/mTOR, JAK/STAT and RTKs (receptor tyrosine kinases) are implicated in the pathogenesis of TNBC cells. The developmental pathways like Wnt/β-Catenin, Notch, Hh (Hedgehog) are associated with invasion, migration, metastatic potential and also self-renewal ability of cancer stem cells. PARP inhibitors directly interact and inhibit molecules associated with DNA repair to increase the cellular damage ultimately leading to apoptosis. Most of the potential inhibitors directly induced apoptosis in TNBC by up-regulation of Bad, Caspase 3 and down-regulation of BCL-2, BCL-XL and survivin. While several inhibitors showed therapeutic response through control of tumour growth and antiproliferative effect on TNBC cells, some inhibitors increased chemosensitivity of TNBC cells. So, synergistic targeting of chemotherapy drugs and therapeutic inhibitors may prove to be an effective way of treatment for TNBC.

Cancer is a network of complex signalling pathways controlled by a cascade of events. Some pathways are highly regulated and are indispensable for the growth, survival, invasion and progression of TNBC. Various pathways are targeted and only a few pathways are found to be sensitive and effective targets for the treatment of TNBC (Figure 1).

NF-κB is a key regulator of inflammatory response, apoptosis and angiogenesis in TNBC and shows four-fold differential expression when compared with normal breast cells [16]. Resistance in cancer cells is developed by abnormal activation of the NF-κB pathway [17]. More than 750 natural and synthetic inhibitors like small molecules, antioxidants, small RNA/DNA, peptides, viral and microbial proteins have been identified as inhibitors of the NF-κB pathway [18]. These inhibitors are used to treat various types of diseases and cancers, but there are no therapeutic drugs for TNBC which may directly interact with NF-κB pathway and thereby treat TNBC. Studies have shown that apoptosis in TNBC is also regulated by the NF-κB pathway. Genistein, a relatively nontoxic and one of the major soy isoflavones, induce apoptosis in TNBC cells by down-regulating the expression of BCL-2, BCL-xL and Cyclin B1 possibly mediated by activation of NF-κB through Notch-1 signalling pathway [19]. Plumbagin inactivates DNA-binding activity of NF-κB and BCL-2 and induces apoptosis in TNBC cells with no effect on normal breast cells [20]. Fenofibrate has antiproliferative effects and induces apoptosis by activation of the NF-κB pathway in TNBC by up-regulation of Bad and activation of Caspase-3, down-regulation of BCL-xL, survivin [21].

JAK/STAT pathway is a key regulator of cellular functions like cell differentiation, proliferation, migration, survival and apoptosis [22]. STAT3 is overexpressed in more than 50% of TNBCs associated with poor prognosis and invasive phenotype [23,24]. Metformin selectively inhibits STAT3 and restricts the growth of the tumour and induces apoptosis in TNBC cells [25]. Ruxolitinib, an inhibitor of JAK1/2 is approved for myelofibrosis treatment [26]. This drug in combination with paclitaxel, doxorubicin and cyclophosphamide is being tested in Phase II clinical trials for triple negative inflammatory breast cancers (Trial Ref.: NCT02876302). In a study, the results showed that *JAK2* gene is amplified in TNBC cells treated with chemotherapy when compared with the tumours before the treatment indicating the JAK2 role in chemoresistance of TNBC. Ruxolitinib failed to inhibit tumour progression in JAK2 amplified TNBC cells. BSK805, a JAK2-specific inhibitor when combined with chemotherapy reduced the tumour growth in mice [27].

PI3K–AKT–mTOR pathway regulates key cellular functions like cell metabolism, proliferation, motility and survival [28]. Almost 60% of TNBCs showed overactivation of PI3K, with its role in deletion or mutation of PTEN tumour suppressor gene. AKT is associated with apoptosis in TNBC by regulating pro-apoptotic molecules like BAD (BCL-2 associated death promoter) [29–31]. AKT activates mTOR through TSC1/2 leading to protein synthesis and cell growth [32]. Activation of PI3K/AKT pathway in ELK3-Knockdown TNBC cells resulted in impaired autophagy and increased chemosensitivity to doxorubicin [33]. Few studies reported that PI3/AKT inhibition increases PARP sensitivity to TNBC cells. PI3K suppression increases sensitivity to PARPi in both BRCA1-deficient and -proficient TNBC patients [31,34]. Buparlisib (PI3K/AKT inhibitor) hyperactivates ERK and MEK1 causing down-regulation of BRCA1. This favours the activity of Olaparib (PARPi) followed by reduction in cancerous cell proliferation [35]. One of the other studies reported that association of Rucaparib (PARPi) and LY294002 (PI3Ki) in BRCA1-deficient cells improves the activity of PARPi [36].

mTOR is a downstream constituent of PI3K/AKT pathway and regulates cellular functions like cell growth, survival, protein turnover and translocation. It exists in two different complexes, mTORC1 and mTORC2. mTORC1 is involved in activation of protein translation and mTORC2 is responsible for AKT phosphorylation. Clinical efficiency of numerous drugs targeting mTOR in TNBC patients is under investigation. Everolimus exhibited antitumour activity in basal-like breast cancer cells in preclinical studies [37]. BEZ235 has shown resistance to the TORC1/2 activity which further activates NOTCH1 that increases population of cancer stem cells. NOTCH activation depends upon FGFR (fibroblast growth factor receptor) 1 (FGFR1)-mitochondrial metabolism. Thus, a combined approach of TORC1/2 inhibitor and FGFR1-mitochondrial metabolism antagonists is required [38]. Some clinical trials have shown that addition of everolimus to paclitaxel in Phase II/III TNBC patients did not show any significant improvement in response ration (RR) and pCR [39–41].

## Role of developmental pathways in TNBC

Wnt/β-catenin signalling plays a major role in embryonic development and tumorigenesis by regulating cell proliferation, differentiation and survival [42–44]. Previous studies reported that aberrant activation of Wnt/β-catenin signalling in TNBC results in poor prognosis [44,45]. Knockdown of β-catenin in TNBC cells significantly decreased cell migration and made TNBC cells more sensitive to chemotherapeutic drugs like cisplatin and doxorubicin [46]. Highly conserved developmental transcription factor SOX4 (sex-determining region Y-box 4) plays a key role in Wnt signalling [47]. SOX4 knockdown has shown to decrease the migration and proliferation in TNBC. Wnt/β-catenin pathway inhibitor ICRT-3 has been reported to inhibit proliferation of TNBC cells [48]. LRP5 and LRP6 of the LDLR (low-density lipoprotein receptor) family are the essential co-receptors for Wnt/β-catenin signalling [43]. LRP6 is overexpressed in TNBC and its knockdown suppresses Wnt/β-catenin signalling *in vivo.* Thus, LRP6 can act as a potential therapeutic target in the treatment of TNBC [49]. To activate Wnt/β-catenin signalling, Wnt binds to both FZD (Frizzled) proteins and LRP5/6. It has been demonstrated that FZD 7 was overexpressed in TNBC and its suppression inactivates Wnt/β-catenin pathway [50]. Secreted glycoproteins like WIF1 and FZD are reported to act as Wnt antagonists. Both the proteins inhibit the interaction of Wnt with FZD receptor hindering the transcription of activated genes by β-catenin/TCF/LEF transcriptional complex [43]. Recently, it has been reported that salinomycin induces degradation of Wnt co-receptor LRP6 [51,52] and also has potential to inhibit the breast cancer cell proliferation [43].

Hh (Hedgehog) signalling dysregulation confers aggressive TNBC phenotype and enhances the invasion, migration and metastatic potential of TNBC cells [53,54]. Previous clinical studies highlighted the key role of Hh signalling in cancer stem cell reprogramming and EMT (epithelial-to-mesenchymal) in TNBC [55,56]. The Hh pathway is associated with embryonic patterning and mediates stem cell renewal by activating the expression of BMI-1, a potent regulator of self-renewal in cancer stem cells [57]. It involves three ligands – IHH (Indian Hedhehog), SHH (Sonic Hedgehog) and DHH (Desert Hedgehog); Transmembrane receptor, PTCH ( Patched) and co-receptor, SMO (Smoothened) [58]. There are three glioma-associated oncogenes (GLI) transcription factors, GLI1, GLI2 and GLI3. However, GLI1 and GLI2 are the most studied ones and responsible for cell proliferation and survival [59]. SMO is the most pharmacologically targeted pathway in TNBC. Various SMO inhibitors were clinically tested and few gave the positive response as Hh antagonists (NCT01071564, NCT02027376 and NCT01757327) [60]. However, in preclinical studies, resistance to these Hh antagonists was observed in TNBC. Thus, a rationale for the GLI-targeted approach was suggested [61]. So far, numerous direct and indirect GLI inhibitors have been clinically tried like GANT61, GANT58 and Glabrescione B (GLaB). These drugs interfere with GLI DNA binding by inhibiting the output of transcription in Hh signalling pathway [62].

The Notch signalling pathway is a much conserved signalling pathway that is mediated by four receptors (NOTCH 1–4) and five ligands (Δ-like 1,3,4 and JAGGED-1,2) [63–66]. Cell–cell contact is a key factor to activate the NOTCH signalling pathway [67]. The signalling cascade is activated by the release of Notch receptor intracellular domain (NICD) with a series of proteolytic cleavage facilitated by γ-secretase [68]. Irregular activation of Notch signalling cascade could initiate malignancies and promote angiogenesis [69]. Previous studies reported that GSI (γ-secretase inhibitors) play a significant role in blocking the Notch signalling pathway [70]. Therefore, numerous preclinical studies have been done on GSI-directed therapy. Researchers confirmed that NOTCH-1 exert a strong influence on tumour proliferation and metastasis. The increased expression of NOTCH-1 has been observed in TNBC that lead to malignancies and poor prognosis [71]. However, it has been recently discovered that NOTCH-4 also plays a pivotal role in the initiation of TNBC and induction of proliferation and tumorigenesis [72]. Targeting NOTCH signalling cascade with GSIs and other drugs should be meticulously explored to increase the survival rate of TNBC patients.

## Receptor-mediated targeting

RTKs (receptor tyrosine kinases) regulate cell growth and metabolism, proliferation and differentiation, cell survival and apoptosis [73]. The therapeutic targets of TNBC in RTK family are VEGFR (vascular endothelial growth factor receptor) [74], PDGFR (platelet-derived growth factor receptor) [75], TGFβR (TGFβ receptor) [76,77], FGFR [78], EGFR (epidermal growth factor receptor) [79,80] and IGF-1R (insulin-like growth factor-1 receptor) [81].

EGFR, also known as HER1 is overexpressed in basal-like cells [80]. EGFR-TKI (tyrosine kinase inhibitor) erlotinib, showed a change in mesenchymal phenotype to epithelial phenotype by up-regulating E-cadherin and down-regulating Vimentin in TNBC cells [82]. Several other EGFR inhibiting agents like panitumumab, cetuximab, gefitinib have shown initial success but failed to produce significant results in clinical studies [83]. Sunitinib is a small-molecule kinase inhibitor, which inhibits both PDGF family and VEGF have shown to reduce tumour volume in xenograft models of TNBC [84]. Bevacizumab reduced progression of metastatic TNBC in 35% of patients in a meta-analysis of Phase III clinical trials [85].

## Epigenetic therapies

It is widely believed that aberrant epigenetic changes in histone deacetylation and DNA hypermethylation may lead to silencing of tumour suppressor genes and drive tumorigenesis in cancer cells [86]. A detailed study of DNA methylation signatures using TCGA (The Cancer Genome Atlas) data helped in the separation of TNBC cells from non-TNBC cells. These data helped in the prognosis of patients by categorizing into poor, medium and good outcomes [87]. The first study showed methylation of a BRCA1 promoter in TNBC and few other studies investigated the role of BRCA1 methylation in TNBC. They also found that BRCA1 methylation increases the sensitivity of TNBC cells towards PARP inhibitors [88]. Another study has found that decreased expression of pRb and increased expression of p76 is associated with BRCA1 [89].

DNA hypermethylation decreases expression of tumour suppressor genes. A study revealed that inhibition of STAT3-DNMT1 (DNA methyltransferase 1) at K685 residue by novel inhibitor SH-I-14 has shown to demethylate the promoter regions of tumour suppressor genes and re-expressed *PDLIM4* and *VHL* genes [90]. A study performed on whole-genome methyl CpG binding domain based capture sequencing (MBDCap-Seq) on TNBC tumours and found 36 differentially methylated regions (DMRs) which showed increased hypermethylation specifically in TNBC cells when compared with non-TNBC samples [91]. BRD4 is a BET (bromodomain and extra terminal) protein family member, regulates mitosis and cell cycle progression [92,93]. BRD4 inhibition has shown to suppress important oncogenic drivers [94]. BETi (BET inhibitor) showed direct inhibition of mitotic regulating proteins AURKA/B in TNBC cells and thereby suppressing tumour growth [95]. BETi JQ1 targeted hypoxic inducing genes and angiogenesis dually in TNBC cells [96]. ID4 (inhibitor of differentiation) protein is highly expressed in TNBC cells and down-regulates BRCA1 pathways [97] and exhibits anchorage-independent growth of breast cancer cells [98]. ID4 promoter hypermethylation is known to increase lymph node metastasis [99]. A study also revealed that ID4 and BRCA1 expression are inversely related and unmethylation of ID4 is associated with BRCAness of breast cancer cells [100]. PKD1 (protein kinase D1) encoded by *PRKD1* gene is abnormally methylated and silenced in invasive breast cancer cells. DNMT inhibitor decitabine reverses PRKD1 promoter methylation and restores PKD1 expression and suppresses lung metastasis in animal models [101].

**Figure 2:**
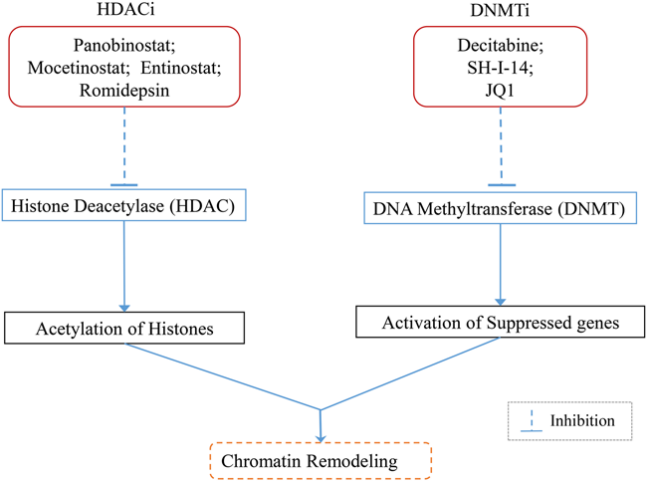
Schematic representation of the mechanism of epigenetic inhibitors in TNBC Aberrant epigenetic changes like histone deacetylation and DNA hypermethylation are associated with silencing of tumour suppressor genes and drive tumorigenesis. Epigenetic inhibitors like Panobinostat; Mocetinostat; Entinostat; Romidepsin inhibit histone deacetylase and promote acetylation of histones leading to transcriptionally active chromatin. DNMT inhibitors Decitabine; SH-I-14; JQ1 help to inhibit DNMT to reactivate suppressed genes.

Another promising epigenetic target for TNBC are HDACi (HDAC inhibitors). HDACi entinostat reduces binding of twist and snail to the CDH-1 promoter, increasing E-cadherin and cytokeratin 8/18 expression and decreasing N-cadherin expression thereby reversing EMT phenotype [102]. Entinostat decreases the expression of CD44^high^/CD24^low^ and markers of TICs (tumour-initiating cells) such as β-catenin, Bmi-1, Nanog, Oct-4 and also reduces mammosphere formation [103]. Romidepsin alone or in combination with paclitaxel removed metastatic lesions and primary tumours in TNBC cells [104]. A potent HDACi Panobinostat decreases cell proliferation, survival, induced apoptosis and inhibits tumour formation in TNBC cells [105]. Another study showed that LBH589 (Panobinostat) inhibits metastasis in TNBC cells mediated by inhibition of ZEB (zinc finger E-box-binding homoeobox) [106] (Figure 2).

Cancer cells disseminate to distant sites by transforming EMT phenotype, which is characterized by loss of E-cadherin expression. TICs which are found in tumour tissues exhibit self-renewing stem cell properties and they also have the ability to grow into a tumour in mice when inoculated at very low numbers [107]. Studies have shown that cancer cells activating EMT acquire TIC’s properties expressing CD44^high^/CD24^low^ markers [108–110].

## Immunotherapies

In 2013, cancer immunotherapy was named as ‘Breakthrough of the year’ by science magazine [111]. TILs (tumour-infiltrating lymphocytes) are long known to be associated with breast cancer prognosis. The prognostic and predictive values vary between subtypes of breast cancer. Studies showed that TILs highly prevailed in TNBC and were less abundant in other types of breast cancer [112]. TILs are prognostic markers for high OS, increased metastasis-free survival and decreased distant recurrence [113,114]. Stromal TILs are correlated with immunological markers like indoleamine 2,3-dioxygenase (IDO1), CD8α, CCL5 (chemokine (C–C motif) ligand 5) and PD-L1 (programmed cell death ligand-1) to significantly increase pCR rates in chemotherapy [115]. Trop-2 (trophoblast cell-surface antigen) is expressed on multiple solid cancers and found to be a novel target for antibody-mediated drug conjugate (ADC) therapy [116]. IMMU-132 is an ADC, delivers topoisomerase-I inhibitor (SN-38) in its most active (non-glucuronidated) form targeting Trop-2 in TNBC [117].

**Figure 3:**
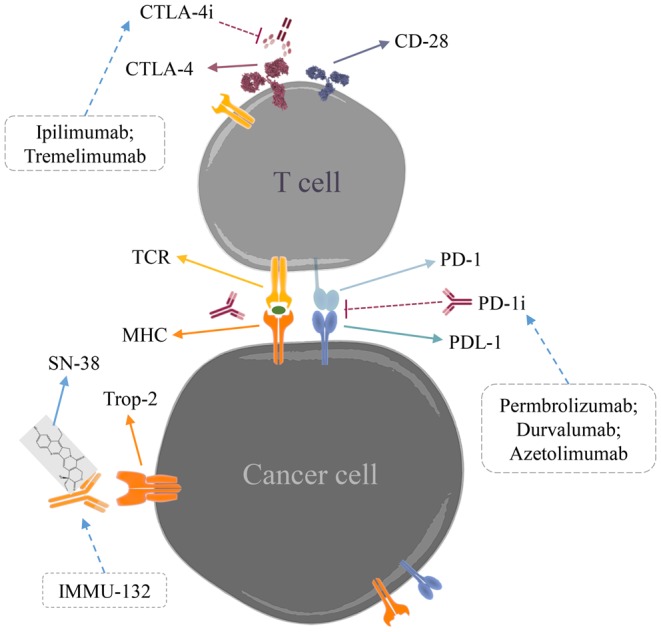
Illustration of immunotherapeutic response in TNBC PD-1 and CTLA-4 are immune checkpoints that prevent immune response towards cancer cells. PD-1 immune checkpoint inhibitors like Permbrolizumab; Durvalumab; Azetolimumab and CTLA-4 inhibitors like Ipilimumab; Tremelimumab inhibit checkpoints and take the ‘brakes off’ T cells thereby activating immune response to attack cancer cells. IMMU-132 is an antibody–drug conjugate delivering topoisomerase I inhibitor (SN-38) targeting Trop-2 receptors on cancer cells.

Immune checkpoints are the molecules of inhibitory pathways in the immune system which play a major role in preventing autoimmunity [118]. Activated CD8^+^ T cells express inhibitory cytotoxic receptor T-lymphocyte associated antigen 4 (CTLA-4), counteracts the activity of co-stimulatory receptor CD28 and attenuates immune response [119]. Ipilimumab is a monoclonal antibody that targets CTLA-4 to activate T cells and thereby increasing proliferation of T cells and potentiates antitumour immune response [120]. Another ‘immune checkpoint’ blockade is PD-1 (programmed cell death 1), a T-cell transmembrane receptor expressed on CD8^+^ T cells. Up-regulation of PD-1 ligands (PD-L1 or PD-L2) blocks T-cell immune response in the tumour microenvironment [121]. Pembrolizumab, a potent inhibitor of PD-1 showed antitumour activity and overall response rate (ORR) of 18.5% in TNBC patients [122] (Figure 3). Other antibodies to take the ‘brakes off’ T cells to increase the antitumour immune response are under investigation and the current immunotherapy clinical trials are listed in Table 1 (Figure 3).

**Table 1:** Recent clinical trials investigating potential therapeutic targets using combinational drug therapy strategy for the treatment of TNBC

Primary drugs	Molecules targeted	Combinatorial drugs	Molecules targeted	Trial reference	Clinical phase	Estimated completion
Everolimus	mTOR	Eribulin	Microtubules	NCT02616848	Phase I	November 2015
MLN0128	mTOR	MLN8237	Aurora A	NCT02719691	Phase I	November 2018
L-NMMA	Nitric oxide synthase	Docetaxel	Microtubules	NCT02834403	Phase I	August 2019
Trilaciclib	CDK4/6 inhibitor	Carboplatin; gemcitabine	DNA damage; nucleosides	NCT02978716	Phase II	December 2019
Ixazomib	Proteasome subunit β-5	Carboplatin	DNA damage	NCT02993094	Phase I/II	September 2019
Selumetinib	MAPK/ERK	Docetaxel; doxobicin; cyclophosphamide	Microtubules; DNA damage	NCT02685657	Phase II	January 2018
Doxorubicin	DNA	Everolimus; bevacizumab	mTOR; VEGF	NCT02456857	Phase II	January 2019
ARQ 092	PI3K/AKT	Carboplatin + paclitaxel/paclitaxel/ anastrozole	DNA damage; tubulin; aromatase	NCT02476955	Phase I	December 2017
Eribulin	Microtubules	PQR309	PI3K/mTOR	NCT02723877	Phase I/II	December 2018
Ruxolitinib	JAK	Paclitaxel; doxobicin; cyclophosphamide	Tubulin; DNA damage	NCT02876302	Phase II	February 2024
Galunisertib	TGF-β	Paclitaxel	Tubulin	NCT02672475	Phase I	January 2020
Vismodegib	SMO (Hh pathway)	Paclitaxel; epirubicin; cyclophosphamide	Tubulin; DNA damage	NCT02694224	Phase II	December 2018
Enzalutamide	Androgen receptor	Paclitaxel	Tubulin	NCT02929576	Phase III	April 2019
Panitumumab	EGFR	Carboplatin; paclitaxel	DNA repair; tubulin	NCT02593175	Phase II	August 2018
Paclitaxel	Tubulin	Afatinib	EGFR	NCT02511847	Phase II	July 2017
Pemetrexed	Nucleotides	Sorafenib	VEGFR, PDGFR	NCT02624700	Phase II	December 2019
Cediranib	VEGF	Olaparib	PARP	NCT02498613	Phase II	May 2018
Cisplatin	DNA damage	Veliparib	PARP	NCT02595905	Phase II	October 2021
Docetaxel	Microtubules	Carboplatin	DNA damage	NCT02547987	Phase II	September 2020
Paclitaxel	Tubulin	Bavituximab	Phosphatidyl-serine	NCT02685306	Phase II	September 2017
Paclitaxel	Tubulin	AT13387	Hsp90	NCT02474173	Phase I	March 2017
Romidepsin	HDAC	Cisplatin	DNA damage	NCT02393794	Phase I/II	December 2018
PDR001	PD-1	LCL161; everolimus or panobinostat	mTOR/HDAC	NCT02890069	Phase I	October 2018
Nivolumab	PD-1	Doxorubicin; cyclophosphamide; cisplatin	DNA damage	NCT02499367	Phase II	August 2022
Pembrolizumab	PD-1	Carboplatin; gemcitabine	DNA damage; nucleosides	NCT02755272	Phase II	April 2023
Pembrolizumab	PD-1	Imprime PGG	B-cell receptor	NCT02981303	Phase II	September 2019
Pembrolizumab	PD-1	Nab-paclitaxel; doxorubicin; cyclophosphamide; carboplatin	Tubulin; DNA damage	NCT02622074	Phase I	August 2017
Pembrolizumab	PD-1	Cyclophosphamide	DNA damage	NCT02768701	Phase II	December 2022
Pembrolizumab	PD-1	Nab-paclitaxel; paclitaxel; gemcitabine; carboplatin	Tubulin; DNA damage; nucleosides	NCT02819518	Phase III	December 2019
Pembrolizumab	PD-1	INCB039110; INCB050465	JAK; PI3K/AKT	NCT02646748	Phase I	December 2017
Pembrolizumab	PD-1	Nab-paclitaxel	Tubulin	NCT02752685	Phase II	December 2018
Eribulin mesylate	Microtubules	Pembrolizumab	PD-1	NCT02513472	Phase I/II	January 2018
Niraparib	PARP	Pembrolizumab	PD-1	NCT02657889	Phase I/II	February 2019
Paclitaxel; capecitabine	Tubulin; nucleotides	Pembrolizumab	PD-1	NCT02734290	Phase I/II	May 2022
Enoblituzumab	B7-H3	Pembrolizumab	PD-1	NCT02475213	Phase I	August 2020
MCS110	M-CSF	PDR001	PD-1	NCT02807844	Phase I/II	February 2019
BLZ945	CSF-1R	PDR001	PD-1	NCT02829723	Phase I/II	June 2019
MPDL3280A	PD-L1	Nab-paclitaxel	Tubulin	NCT02530489	Phase II	February 2021
MPDL3280A	PD-L1	Carboplatin; abraxane	DNA damage; Tubulin	NCT02620280	Phase III	May 2022
Durvalumab	PD-L1	Vigil	T cells	NCT02725489	Phase II/III	May 2018
Durvalumab	PD-L1	Nab-paclitaxel; epirubicin; cyclophosphamide	Tubulin; DNA damage	NCT02685059	Phase II	March 2018
Durvalumab	PD-L1	Olaparib; cediranib	PARP; VEGF	NCT02484404	Phase I/II	December 2019
Atezolizumab	PD-L1	Carboplatin; paclitaxel	DNA damage; tubulin	NCT02883062	Phase II	September 2019
Veliparib	PARP	Atezolizumab	PD-L1	NCT02849496	Phase II	August 2018
Nab-paclitaxel	Tubulin	Atezolizumab	PD-L1	NCT02425891	Phase III	April 2020
Entinostat	HDAC	Atezolizumab	PD-L1	NCT02708680	Phase I/II	June 2019
Varlilumab	CD-27	Atezolizumab	PD-L1	NCT02543645	Phase I/II	June 2019
Nab-paclitaxel	Tubulin	MPD3280A	PD-L1	NCT02530489	Phase II	February 2021
Durvalumab	PD-L1	Nab-paclitaxel; dose-dense doxorubicin/cyclophosphamide	Tubulin; DNA/RNA damage	NCT02489448	Phase I/II	October 2019
Tremelimumab	CTLA-4	Durvalumab	PD-L1	NCT02527434	Phase II	April 2018
Enoblituzumab	B7-H3	Ipilimumab	CTLA-4	NCT02381314	Phase I	March 2018
Carboplatin; Gemcitabine	DNA damage; nucleosides	MCS110	M-CSF	NCT02435680	Phase II	March 2019

Details provided in the table include only recent clinical trials which are first received on or after 01/01/2015.

## Combined drug therapy strategies

Although the single-agent therapy has shown positive results in cell lines and preclinical models but failed to get promising results in clinical trials to counter aggressive TNBC, owing to its heterogeneity and acquired drug resistance. Combined drug therapy (CDT) is rapidly gaining popularity and proving to be effective in current clinical trials towards improving pCR, PFS and OS in various cancers. At present, almost 80% of the clinical trials are using combinatorial drugs to investigate new therapeutic strategies for TNBC treatment. CDT strategies in current clinical trials data are provided (Table 1).

Recently, CDT strategy has been widely used for immunotherapy checkpoint inhibitors to target TNBC effectively. Tremelimumab (CTLA-4i) in combination with duralumin (PD-L1i) is under investigation in Phase II clinical trials (NCT02527434). The effective way of planning combinational strategy is through prediction of effective targets connected to signalling networks that drive cancer progression. Systems biology provided attractive tools to strategize network-based therapies for cancer. Using these tools, a study group identified five most effective and connected targets (VIM, YWHAB, TK1, CSNK2B and HSP90AB1) in TNBC cells. Initially, the targets were validated using cell-based assays. Based on initial results, using animal models they knocked out five targets *in vivo* and successfully inhibited colony formation, proliferation, migration, anchorage independence and invasion [123].

A study showed that combination of mTOR inhibitor rapamycin and doxorubicin-loaded cyclic octapeptide liposomes inhibited the expression of HIF-1α in TNBC cells [124]. Combined inhibition of PI3K/AKT/mTOR with chemotherapy showed substantial improvement in PFS of TNBC patients [125]. Other study showed that combined inhibition of CDK4/6 and PI3Kα has greatly increased tumour infiltrating T-cell activation in TNBC cells [126]. TNBC cells which expressed PTEN responded to PARP and HDACis. Combined inhibition of olaparib and SAHA in TNBC cells showed increased DNA damage, decreased proliferation, increased autophagy and apoptosis [127]. HDACi mocetinostat combinedly treated with BETi JQ1 showed synergistic suppression of cell cycle progression genes and induced apoptosis in TNBC cells [128].

Few randomized clinical trials showed that addition of HDACi to DNMTi did not improve the outcomes in the patients [129–131]. There is no conclusive evidence that epigenetic inhibitors function by epigenetic mechanisms. These results clearly indicate to reinvestigate how epigenetic drugs work and their mechanism of action [132].

## Future directions

The recent study shows that knockdown of PRL-3 (phosphatase of regenerating liver 3) leads cancer cells to senescence. The experimental drug AMPI-109 inactivates PRL-3, making senescent cancer cells sensitive for immunotherapy treatment [133].

There is an increasing evidence indicating the role of PTEN in acquiring chemoresistance in MDR (multidrug resistant) breast cancer cells. Inhibition of *miR-19* down-regulates multidrug resistance genes (*MDR-1, MRP-1* and *BCRP*) and restores PTEN expression in MDR breast cancer cells, sensitizing cells to chemotherapeutic agents [134]. Up-regulation of PTEN activity increases the effectiveness of chemotherapy and in combination with ID4 (DNA binding protein inhibitor) can be studied for the effective treatment of TNBC. One of the studies suggested that combination therapy of lapatinib (NF-κB inhibitor) with a proteasome inhibitor may prove to be an effective treatment for TNBC [135].

A study published in 2011, shows that anti-oestrogens or aromatase inhibitors increase the population of ER-negative cells in luminal breast cancer cells thereby increasing resistance to the treatment [136]. This study led to the findings that inhibiting Notch-1 in luminal breast cancers maintains the ER positive state for the effective targeting of ER-based therapies. It is also found that inhibiting Notch-1 can transform ER−/PR−/CK5+ cells to ER+ cells [137]. Therefore, Notch-1 inhibitors like GSI in combination with endocrine therapies can be used as CDT strategy for TNBC treatment.

Several other chemotherapy drugs, epigenetic inhibitors, immunotherapies and combinational therapies showing positive results *in vitro* should be immediately carried over to clinical trials to determine the effectiveness of the drugs *in vivo*. As there is an urgent need to find out therapeutic targets for TNBC, we need to explore the new biomarkers and signalling pathways which help in early diagnosis of cancer and finding new therapeutic targets for effective treatment of TNBC.

## Conclusion

Despite the fact that combined therapeutic strategies are proven to be effective in various cancers including TNBC, there are few exemptions where some of the valid hypotheses and *in vitro* results are shown to be ineffective when translated into clinical trials. TNBC is a heterogeneous cancer with varying physiological and pathological characteristics and associated with the aggressive phenotype. So, despite the emergence of various therapeutic strategies for the treatment of TNBC, the effective treatment can be provided by selecting suitable combinational therapy by considering patient-specific molecular characteristics, biomarkers, clinical and pathological features through proper diagnosis.

## Abbreviations

BET: bromodomain and extra terminal
BETi: BET inhibitor
CDT: combined drug therapy
CTLA-4: cytotoxic receptor T-lymphocyte associated antigen 4
DNMT: DNA methyltransferase
EGFR: epidermal growth factor receptor
EMT: epithelial-to-mesenchymal
FGFR: fibroblast growth factor receptor
FZD: frizzled protein
GLI: glioma-associated oncogene
GSI: γ-secretase inhibitor
HDACi: HDAC inhibitor
Hh: hedgehog
ID4: inhibitor of differentiation
MDR: multidrug resistant
OS: overall survival
pCR: pathological clinical response
PD-1: programmed cell death 1
PD-L1: programmed cell death ligand-1
PFS: progression-free survival
PKD1: protein kinase D1
PRL-3: phosphatase of regenerating liver 3
RTK: receptor tyrosine kinase
SMO: smoothened
SOX4: sex-determining region Y-box 4
TIC: tumour-initiating cell
TIL: tumour-infiltrating lymphocyte
TNBC: triple negative breast cancer
Trop-2: trophoblast cell-surface antigen

## References

[1] Ferlay J. (2015) Cancer incidence and mortality worldwide: sources, methods and major patterns in GLOBOCAN 2012. Int. J. Cancer 136, E359–E386 10.1002/ijc.29210 25220842

[2] Gajulapalli V.N.R. (2016) Oestrogen receptor negativity in breast cancer: a cause or consequence ? Biosci. Rep. 36, e00432 10.1042/BSR20160228 27884978 PMC5180249

[3] Polyak K. (2011) Heterogeneity in breast cancer. J. Clin. Invest. 121, 3786 10.1172/JCI60534 21965334 PMC3195489

[4] Kaur P. (2012) A mouse model for triple-negative breast cancer tumor-initiating cells (TNBC-TICs) exhibits similar aggressive phenotype to the human disease. BMC Cancer 12, 120 10.1186/1471-2407-12-120 22452810 PMC3340297

[5] Irvin W.J. and Carey L.A. (2008) What is triple-negative breast cancer? Eur. J. Cancer 44, 2799–2805 10.1016/j.ejca.2008.09.034 19008097

[6] Blows F.M. (2010) Subtyping of breast cancer by immunohistochemistry to investigate a relationship between subtype and short and long term survival: a collaborative analysis of data for 10,159 cases from 12 studies. PLoS Med. 7, e1000279 10.1371/journal.pmed.1000279 20520800 PMC2876119

[7] Sharma P. (2016) Biology and management of patients with triple-negative breast cancer. Oncologist 21, 10.1634/theoncologist.2016-0067 PMC5016071 27401886

[8] Lips E. (2013) Triple-negative breast cancer: BRCAness and concordance of clinical features with BRCA1-mutation carriers. Br. J. Cancer 108, 2172–2177 10.1038/bjc.2013.144 23558900 PMC3670471

[9] Sharma P. (2014) The prognostic value of BRCA1 promoter methylation in early stage triple negative breast cancer. J. Cancer Ther. Res. 3, 1–11 10.7243/2049-7962-3-2 25177489 PMC4147783

[10] Sharma P. (2014) Germline BRCA mutation evaluation in a prospective triple-negative breast cancer registry: implications for hereditary breast and/or ovarian cancer syndrome testing. Breast Cancer Res. Treat. 145, 707–714 10.1007/s10549-014-2980-0 24807107 PMC4171847

[11] Gonzalez-Angulo A.M. (2011) Incidence and outcome of BRCA mutations in unselected patients with triple receptor-negative breast cancer. Clin. Cancer Res. 17, 1082–1089 10.1158/1078-0432.CCR-10-2560 21233401 PMC3048924

[12] Hartman A.R. (2012) Prevalence of BRCA mutations in an unselected population of triple‐negative breast cancer. Cancer 118, 2787–2795 10.1002/cncr.26576 22614657

[13] Lehmann B.D. (2011) Identification of human triple-negative breast cancer subtypes and preclinical models for selection of targeted therapies. J. Clin. Invest. 121, 2750–2767 10.1172/JCI45014 21633166 PMC3127435

[14] Kreike B. (2007) Gene expression profiling and histopathological characterization of triple-negative/basal-like breast carcinomas. Breast Cancer Res. 9, R65 10.1186/bcr1771 17910759 PMC2242660

[15] Carey L.A. (2006) Race, breast cancer subtypes, and survival in the Carolina Breast Cancer Study. JAMA 295, 2492–2502 10.1001/jama.295.21.2492 16757721

[16] Ossovskaya V. (2011) Exploring molecular pathways of triple-negative breast cancer. Genes Cancer 2, 870–879 10.1177/1947601911432496 22593799 PMC3352156

[17] Fan Y. (2008) Regulation of programmed cell death by NF-κB and its role in tumorigenesis and therapy. In Programmed Cell Death in Cancer Progression and Therapy, pp. 223–250, Springer 10.1007/978-1-4020-6554-5_11 18437897

[18] Gilmore T. and Herscovitch M. (2006) Inhibitors of NF-κB signaling: 785 and counting. Oncogene 25, 6887–6899 10.1038/sj.onc.1209982 17072334

[19] Pan H. (2012) Genistein inhibits MDA-MB-231 triple-negative breast cancer cell growth by inhibiting NF-κB activity via the Notch-1 pathway. Int. J. Mol. Med. 30, 337–343 10.3892/ijmm.2012.990 22580499

[20] Ahmad A. (2008) Plumbagin‐induced apoptosis of human breast cancer cells is mediated by inactivation of NF‐κB and Bcl‐2. J. Cell. Biochem. 105, 1461–1471 10.1002/jcb.21966 18980240

[21] Li T. (2014) Fenofibrate induces apoptosis of triple-negative breast cancer cells via activation of NF-κB pathway. BMC Cancer 14, 96 24529079 10.1186/1471-2407-14-96 PMC4015735

[22] Furth P.A. (2014) STAT signaling in different breast cancer sub-types. Mol. Cell. Endocrinol. 382, 612–615 10.1016/j.mce.2013.03.023 23562422 PMC3740183

[23] Wei W. (2014) STAT3 signaling is activated preferentially in tumor‐initiating cells in claudin‐low models of human breast cancer. Stem Cell 32, 2571–2582 10.1002/stem.1752 24891218

[24] Shields B.J. (2013) TCPTP regulates SFK and STAT3 signaling and is lost in triple-negative breast cancers. Mol. Cell. Biol. 33, 557–570 10.1128/MCB.01016-12 23166300 PMC3554209

[25] Deng X.-S. (2012) Metformin targets Stat3 to inhibit cell growth and induce apoptosis in triple-negative breast cancers. Cell Cycle 11, 367–376 10.4161/cc.11.2.18813 22189713

[26] Harrison C. (2015) JAK inhibitors and myelofibrosis, Einstein and ruxolitinib. Haematologica 100, 409–411 10.3324/haematol.2015.124099 25828084 PMC4380710

[27] Balko J.M. (2016) Triple-negative breast cancers with amplification of JAK2 at the 9p24 locus demonstrate JAK2-specific dependence. Sci. Transl. Med. 8, 334ra353 10.1126/scitranslmed.aad3001 PMC5256931 27075627

[28] Massihnia D. (2016) Triple negative breast cancer: shedding light onto the role of pi3k/akt/mtor pathway. Oncotarget 10.18632/oncotarget.10858 27474173 PMC5312414

[29] Gordon V. and Banerji S. (2013) Molecular pathways: PI3K pathway targets in triple-negative breast cancers. Clin. Cancer Res. 19, 3738–3744 10.1158/1078-0432.CCR-12-0274 23748695

[30] Gewinner C. (2009) Evidence that inositol polyphosphate 4-phosphatase type II is a tumor suppressor that inhibits PI3K signaling. Cancer Cell 16, 115–125 10.1016/j.ccr.2009.06.006 19647222 PMC2957372

[31] Fruman D.A. and Rommel C. (2014) PI3K and cancer: lessons, challenges and opportunities. Nat. Rev. Drug Discov. 13, 140–156 10.1038/nrd4204 24481312 PMC3994981

[32] Lauring J. (2013) The phosphoinositide-3-kinase-Akt-mTOR pathway as a therapeutic target in breast cancer. J. Natl. Compr. Cancer Netw. 11, 670–678 10.6004/jnccn.2013.0086 23744866 PMC4086482

[33] Park J.-H. (2016) PI3K/Akt/mTOR activation by suppression of ELK3 mediates chemosensitivity of MDA-MB-231 cells to doxorubicin by inhibiting autophagy. Biochem. Biophys. Res. Commun., 10.1016/j.bbrc.2016.06.057 27301639

[34] Juvekar A. (2012) Combining a PI3K inhibitor with a PARP inhibitor provides an effective therapy for BRCA1-related breast cancer. Cancer Discov. 2, 1048–1063 10.1158/2159-8290.CD-11-0336 22915751 PMC3733368

[35] Ibrahim Y.H. (2012) PI3K inhibition impairs BRCA1/2 expression and sensitizes BRCA-proficient triple-negative breast cancer to PARP inhibition. Cancer Discov. 2, 1036–1047 10.1158/2159-8290.CD-11-0348 22915752 PMC5125254

[36] Kimbung S. (2012) Co-targeting of the PI3K pathway improves the response of BRCA1 deficient breast cancer cells to PARP1 inhibition. Cancer Lett. 319, 232–241 10.1016/j.canlet.2012.01.015 22266096

[37] Yunokawa M. (2012) Efficacy of everolimus, a novel mTOR inhibitor, against basal‐like triple‐negative breast cancer cells. Cancer Sci. 103, 1665–1671 10.1111/j.1349-7006.2012.02359.x 22703543 PMC7659327

[38] Bhola N.E. (2016) Treatment of triple-negative breast cancer with TORC1/2 inhibitors sustains a drug-resistant and notch-dependent cancer stem cell population. Cancer Res. 76, 440–452 10.1158/0008-5472.CAN-15-1640-T 26676751 PMC4715956

[39] Mayer I. (2013) Abstract PD1-6: a randomized phase II neoadjuvant study of cisplatin, paclitaxel with or without everolimus (an mTOR inhibitor) in patients with stage II/III triple-negative breast cancer (TNBC). Cancer Res. 73, PD1–6 10.1158/0008-5472.SABCS13-PD1-6

[40] Gonzalez-Angulo A. (2014) Open-label randomized clinical trial of standard neoadjuvant chemotherapy with paclitaxel followed by FEC versus the combination of paclitaxel and everolimus followed by FEC in women with triple receptor-negative breast cancer. Ann. Oncol. 25, 1122–1127 10.1093/annonc/mdu124 24669015 PMC4037860

[41] Jerusalem G. (2014) Use of mTOR inhibitors in the treatment of breast cancer: an evaluation of factors that influence patient outcomes. Breast Cancer 6, 43–57 24833916 10.2147/BCTT.S38679 PMC4000187

[42] Howe L.R. and Brown A.M. (2004) Wnt signaling and breast cancer. Cancer Biol. Ther. 3, 36–41 10.4161/cbt.3.1.561 14739782

[43] MacDonald B.T. (2009) Wnt/β-catenin signaling: components, mechanisms, and diseases. Dev. Cell 17, 9–26 10.1016/j.devcel.2009.06.016 19619488 PMC2861485

[44] Khramtsov A.I. (2010) Wnt/β-catenin pathway activation is enriched in basal-like breast cancers and predicts poor outcome. Am. J. Pathol. 176, 2911–2920 10.2353/ajpath.2010.091125 20395444 PMC2877852

[45] Barker N. and Clevers H. (2006) Mining the Wnt pathway for cancer therapeutics. Nat. Rev. Drug Discov. 5, 997–1014 10.1038/nrd2154 17139285

[46] Xu J. (2015) β-Catenin is required for the tumorigenic behavior of triple-negative breast cancer cells. PLoS ONE 10, e0117097 10.1371/journal.pone.0117097 25658419 PMC4319896

[47] Scharer C.D. (2009) Genome-wide promoter analysis of the SOX4 transcriptional network in prostate cancer cells. Cancer Res. 69, 709–717 10.1158/0008-5472.CAN-08-3415 19147588 PMC2629396

[48] Bilir B. (2013) Wnt signaling blockage inhibits cell proliferation and migration, and induces apoptosis in triple-negative breast cancer cells. J. Transl. Med. 11, 280 10.1186/1479-5876-11-280 24188694 PMC4228255

[49] Liu C.-C. (2010) LRP6 overexpression defines a class of breast cancer subtype and is a target for therapy. Proc. Natl. Acad. Sci. U.S.A. 107, 5136–5141 10.1073/pnas.0911220107 20194742 PMC2841938

[50] Yang L. (2011) FZD7 has a critical role in cell proliferation in triple negative breast cancer. Oncogene 30, 4437–4446 10.1038/onc.2011.145 21532620

[51] Lu D. (2011) Salinomycin inhibits Wnt signaling and selectively induces apoptosis in chronic lymphocytic leukemia cells. Proc. Natl. Acad. Sci. U.S.A. 108, 13253–13257 10.1073/pnas.1110431108 21788521 PMC3156152

[52] Tang Q.-L. (2011) Salinomycin inhibits osteosarcoma by targeting its tumor stem cells. Cancer Lett. 311, 113–121 10.1016/j.canlet.2011.07.016 21835542

[53] Kwon Y.-J. (2011) Gli1 enhances migration and invasion via up-regulation of MMP-11 and promotes metastasis in ERα negative breast cancer cell lines. Clin. Exp. Metastasis 28, 437–449 10.1007/s10585-011-9382-z 21442356 PMC3081062

[54] Harris L.G. (2012) Increased vascularity and spontaneous metastasis of breast cancer by hedgehog signaling mediated upregulation of cyr61. Oncogene 31, 3370–3380 10.1038/onc.2011.496 22056874 PMC3276742

[55] Lei J. (2015) Gli-1 is crucial for hypoxia-induced epithelial-mesenchymal transition and invasion of breast cancer. Tumour Biol. 36, 3119–3126 10.1007/s13277-014-2948-z 25501705

[56] Sims‐Mourtada J. (2015) Taxane‐induced hedgehog signaling is linked to expansion of breast cancer stem‐like populations after chemotherapy. Mol. Carcinog. 54, 1480–1493 10.1002/mc.22225 25263583

[57] Yang N. (2016) Inhibition of sonic hedgehog signaling pathway by thiazole antibiotic thiostrepton attenuates the CD44+/CD24-stem-like population and sphere-forming capacity in triple-negative breast cancer. Cell. Physiol. Biochem. 38, 1157–1170 10.1159/000443066 26963129

[58] Sasai N. and Briscoe J. (2012) Primary cilia and graded Sonic Hedgehog signaling. Wiley Interdiscip. Rev. Dev. Biol. 1, 753–772 10.1002/wdev.43 23799571

[59] Alexandre C. (1996) Transcriptional activation of hedgehog target genes in *Drosophila* is mediated directly by the cubitus interruptus protein, a member of the GLI family of zinc finger DNA-binding proteins. Genes Dev. 10, 2003–2013 10.1101/gad.10.16.2003 8769644

[60] Merchant A.A. and Matsui W. (2010) Targeting Hedgehog—a cancer stem cell pathway. Clin. Cancer Res. 16, 3130–3140 10.1158/1078-0432.CCR-09-2846 20530699 PMC2888641

[61] Kameda C. (2009) The Hedgehog pathway is a possible therapeutic target for patients with estrogen receptor-negative breast cancer. Anticancer Res. 29, 871–879 19414322

[62] Lauth M. (2007) Inhibition of GLI-mediated transcription and tumor cell growth by small-molecule antagonists. Proc. Natl. Acad. Sci. U.S.A. 104, 8455–8460 10.1073/pnas.0609699104 17494766 PMC1866313

[63] Lindsell C.E. (1995) Jagged: a mammalian ligand that activates Notch1. Cell 80, 909–917 10.1016/0092-8674(95)90294-5 7697721

[64] Shawber C. (1996) Jagged2: a serrate-like gene expressed during rat embryogenesis. Dev. Biol. 180, 370–376 10.1006/dbio.1996.0310 8948600

[65] Speiser J. (2012) Notch-1 and Notch-4 biomarker expression in triple-negative breast cancer. Int. J. Surg. Pathol. 20, 137–143 10.1177/1066896911427035 22084425

[66] Blaumueller C. and Artavanis-Tsakonas S. (1996) Comparative aspects of Notch signaling in lower and higher eukaryotes. Perspect. Dev. Neurobiol. 4, 325–343 9171446

[67] Callahan R. and Raafat A. (2001) Notch signaling in mammary gland tumorigenesis. J. Mammary Gland Biol. Neoplasia 6, 23–36 10.1023/A:1009512414430 11467450

[68] Bolós V. (2007) Notch signaling in development and cancer. Endocr. Rev. 28, 339–363 10.1210/er.2006-0046 17409286

[69] Phng L.-K. and Gerhardt H. (2009) Angiogenesis: a team effort coordinated by notch. Dev. Cell 16, 196–208 10.1016/j.devcel.2009.01.015 19217422

[70] Li Z.-L. (2015) Gamma secretase inhibitor enhances sensitivity to doxorubicin in MDA-MB-231 cells. Int. J. Clin. Exp. Pathol. 8, 4378–4387 26191129 PMC4503001

[71] Bolós V. (2013) Notch activation stimulates migration of breast cancer cells and promotes tumor growth. Breast Cancer Res. 15, R54 10.1186/bcr3447 23826634 PMC3978930

[72] Nagamatsu I. (2014) NOTCH4 is a potential therapeutic target for triple-negative breast cancer. Anticancer Res. 34, 69–80 24403446

[73] Zhang J. and Hochwald S.N. (2013) Targeting receptor tyrosine kinases in solid tumors. Surg. Oncol. Clin. N. Am. 22, 685–703 10.1016/j.soc.2013.06.010 24012395

[74] Dent S. (2009) The role of VEGF in triple-negative breast cancer: where do we go from here? Ann. Oncol. 20, 1615–1617 10.1093/annonc/mdp410 19690059

[75] Carvalho I. (2005) Overexpression of platelet-derived growth factor receptor α in breast cancer is associated with tumour progression. Breast Cancer Res. 7, R788–R795 10.1186/bcr1304 16168125 PMC1242156

[76] Bhola N.E. (2013) TGF-β inhibition enhances chemotherapy action against triple-negative breast cancer. J. Clin. Invest. 123, 1348–1358 10.1172/JCI65416 23391723 PMC3582135

[77] Jovanović B. (2014) Transforming growth factor beta receptor type III is a tumor promoter in mesenchymal-stem like triple negative breast cancer. Breast Cancer Res. 16, R69 10.1186/bcr3684 24985072 PMC4095685

[78] Sharpe R. (2011) FGFR signaling promotes the growth of triple-negative and basal-like breast cancer cell lines both *in vitro* and *in vivo*. Clin. Cancer Res. 17, 5275–5286 10.1158/1078-0432.CCR-10-2727 21712446 PMC3432447

[79] Song H. (2013) Targeting aberrant DNA double-strand break repair in triple-negative breast cancer with alpha-particle emitter radiolabeled anti-EGFR antibody. Mol. Cancer Ther. 12, 2043–2054 10.1158/1535-7163.MCT-13-0108 23873849 PMC3804319

[80] Corkery B. (2009) Epidermal growth factor receptor as a potential therapeutic target in triple-negative breast cancer. Ann. Oncol. 20, 862–867 10.1093/annonc/mdn710 19150933

[81] Litzenburger B.C. (2011) High IGF-IR activity in triple-negative breast cancer cell lines and tumorgrafts correlates with sensitivity to anti–IGF-IR therapy. Clin. Cancer Res. 17, 2314–2327 10.1158/1078-0432.CCR-10-1903 21177763 PMC3073089

[82] Ueno N.T. and Zhang D. (2011) Targeting EGFR in triple negative breast cancer. J. Cancer 2, 324–328 10.7150/jca.2.324 21716849 PMC3119395

[83] Amin D.N. (2010) Resiliency and vulnerability in the HER2-HER3 tumorigenic driver. Sci. Transl. Med. 2, 16ra17–16ra17 10.1126/scitranslmed.3000389 PMC3033659 20371474

[84] Chinchar E. (2014) Sunitinib significantly suppresses the proliferation, migration, apoptosis resistance, tumor angiogenesis and growth of triple-negative breast cancers but increases breast cancer stem cells. Vasc. Cell 6, 12 10.1186/2045-824X-6-12 24914410 PMC4049452

[85] O’Shaughnessy J. (2010) Abstract P6-12-03: meta-analysis of patients with triple-negative breast cancer (TNBC) from three randomized trials of first-line bevacizumab (BV) and chemotherapy treatment for metastatic breast cancer (MBC). Cancer Res. 70, 10.1158/0008-5472.SABCS10-P6-12-03

[86] Jones P.A. and Baylin S.B. (2007) The epigenomics of cancer. Cell 128, 683–692 10.1016/j.cell.2007.01.029 17320506 PMC3894624

[87] Stirzaker C. (2015) Methylome sequencing in triple-negative breast cancer reveals distinct methylation clusters with prognostic value. Nat. Commun. 6, 10.1038/ncomms6899 25641231

[88] Veeck J. (2010) BRCA1 CpG island hypermethylation predicts sensitivity to poly (adenosine diphosphate)-ribose polymerase inhibitors. J. Clin. Oncol. 28, e563–e564 10.1200/JCO.2010.30.1010 20679605

[89] Stefansson O.A. (2011) CpG island hypermethylation of BRCA1 and loss of pRb as co-occurring events in basal/triple-negative breast cancer. Epigenetics 6, 638–649 10.4161/epi.6.5.15667 21593597 PMC3121973

[90] Kang H.J. (2015) Disruption of STAT3-DNMT1 interaction by SH-I-14 induces re-expression of tumor suppressor genes and inhibits growth of triple-negative breast tumor. Oncotarget 5, 10.18632/oncotarget.4054 PMC5663528 29137356

[91] Stirzaker C. (2016) Genome-wide DNA methylation profiling in triple-negative breast cancer reveals epigenetic signatures with important clinical value. Mol. Cell. Oncol. 3, e1038424 10.1080/23723556.2015.1038424 27308556 PMC4845196

[92] Wu S.-Y. and Chiang C.-M. (2007) The double bromodomain-containing chromatin adaptor Brd4 and transcriptional regulation. J. Biol. Chem. 282, 13141–13145 10.1074/jbc.R700001200 17329240

[93] Dey A. (2000) A bromodomain protein, MCAP, associates with mitotic chromosomes and affects G2-to-M transition. Mol. Cell. Biol. 20, 6537–6549 10.1128/MCB.20.17.6537-6549.2000 10938129 PMC86127

[94] Lovén J. (2013) Selective inhibition of tumor oncogenes by disruption of super-enhancers. Cell 153, 320–334 10.1016/j.cell.2013.03.036 23582323 PMC3760967

[95] Sahni J.M. (2016) Bromodomain and extraterminal protein inhibition blocks growth of triple-negative breast cancers through the suppression of Aurora kinases. J. Biol. Chem. 10.1074/jbc.M116.738666 PMC5095428 27650498

[96] da Motta L. (2016) The BET inhibitor JQ1 selectively impairs tumour response to hypoxia and downregulates CA9 and angiogenesis in triple negative breast cancer. Oncogene, 10.1038/onc.2016.184 27292261 PMC5061082

[97] Wen Y.H. (2012) Id4 protein is highly expressed in triple-negative breast carcinomas: possible implications for BRCA1 downregulation. Breast Cancer Res. Treat. 135, 93–102 10.1007/s10549-012-2070-0 22538771

[98] Beger C. (2001) Identification of Id4 as a regulator of BRCA1 expression by using a ribozyme-library-based inverse genomics approach. Proc. Natl. Acad. Sci. U.S.A. 98, 130–135 10.1073/pnas.98.1.130 11136250 PMC14556

[99] Umetani N. (2005) Aberrant hypermethylation of ID4 gene promoter region increases risk of lymph node metastasis in T1 breast cancer. Oncogene 24, 4721–4727 10.1038/sj.onc.1208538 15897910

[100] Branham M. (2016) Epigenetic regulation of ID4 in the determination of the BRCAness phenotype in breast cancer. Breast Cancer Res. Treat. 155, 13–23 10.1007/s10549-015-3648-0 26610810 PMC6036618

[101] Borges S. (2013) Pharmacologic reversion of epigenetic silencing of the PRKD1 promoter blocks breast tumor cell invasion and metastasis. Breast Cancer Res. 15, R66 10.1186/bcr3460 23971832 PMC4052945

[102] Shah P. (2014) Histone deacetylase inhibitor entinostat reverses epithelial to mesenchymal transition of breast cancer cells by reversing the repression of E-cadherin. Breast Cancer Res. Treat. 143, 99–111 10.1007/s10549-013-2784-7 24305977

[103] Schech A. (2015) Histone deacetylase inhibitor entinostat inhibits tumor-initiating cells in triple-negative breast cancer cells. Mol. Cancer Ther. 14, 1848–1857 10.1158/1535-7163.MCT-14-0778 26037781

[104] Robertson F.M. (2013) The class I HDAC inhibitor Romidepsin targets inflammatory breast cancer tumor emboli and synergizes with paclitaxel to inhibit metastasis. J. Exp. Ther. Oncol. 10, 219–233 24416998

[105] Tate C.R. (2012) Targeting triple-negative breast cancer cells with the histone deacetylase inhibitor panobinostat. Breast Cancer Res. 14, R79 10.1186/bcr3192 22613095 PMC3446342

[106] Rhodes L.V. (2014) Suppression of triple-negative breast cancer metastasis by pan-DAC inhibitor panobinostat via inhibition of ZEB family of EMT master regulators. Breast Cancer Res. Treat. 145, 593–604 10.1007/s10549-014-2979-6 24810497 PMC4083690

[107] Reya T. (2001) Stem cells, cancer, and cancer stem cells. Nature 414, 105–111 10.1038/35102167 11689955

[108] Morel A.-P. (2008) Generation of breast cancer stem cells through epithelial-mesenchymal transition. PLoS ONE 3, e2888 10.1371/journal.pone.0002888 18682804 PMC2492808

[109] Mani S.A. (2008) The epithelial-mesenchymal transition generates cells with properties of stem cells. Cell 133, 704–715 10.1016/j.cell.2008.03.027 18485877 PMC2728032

[110] Scheel C. and Weinberg R.A. (2012) Cancer stem cells and epithelial–mesenchymal transition: concepts and molecular links, Semin. Cancer Biol. 22, 396–403 22554795 10.1016/j.semcancer.2012.04.001 PMC6220425

[111] Couzin-Frankel J. (2013) Cancer immunotherapy. Science 342, 1432–1433 10.1126/science.342.6165.1432 24357284

[112] Pusztai L. (2016) New strategies in breast cancer: immunotherapy. Clin. Cancer Res. 22, 2105–2110 10.1158/1078-0432.CCR-15-1315 26867935 PMC9359478

[113] Loi S. (2014) Tumor infiltrating lymphocytes are prognostic in triple negative breast cancer and predictive for trastuzumab benefit in early breast cancer: results from the FinHER trial. Ann. Oncol. 25, 1544–1550 10.1093/annonc/mdu112 24608200

[114] Adams S. (2014) Prognostic value of tumor-infiltrating lymphocytes in triple-negative breast cancers from two phase III randomized adjuvant breast cancer trials: ECOG 2197 and ECOG 1199. J. Clin. Oncol. 32, 2959–2966 10.1200/JCO.2013.55.0491 25071121 PMC4162494

[115] Denkert C. (2015) Tumor-infiltrating lymphocytes and response to neoadjuvant chemotherapy with or without carboplatin in human epidermal growth factor receptor 2–positive and triple-negative primary breast cancers. J. Clin. Oncol. 33, 983–991 10.1200/JCO.2014.58.1967 25534375

[116] Cardillo T.M. (2011) Humanized anti-Trop-2 IgG-SN-38 conjugate for effective treatment of diverse epithelial cancers: preclinical studies in human cancer xenograft models and monkeys. Clin. Cancer Res. 17, 3157–3169 10.1158/1078-0432.CCR-10-2939 21372224 PMC10766325

[117] Goldenberg D.M. (2015) Trop-2 is a novel target for solid cancer therapy with sacituzumab govitecan (IMMU-132), an antibody-drug conjugate (ADC). Oncotarget 6, 22496 10.18632/oncotarget.4318 26101915 PMC4673178

[118] Pardoll D.M. (2012) The blockade of immune checkpoints in cancer immunotherapy. Nat. Rev. Cancer 12, 252–264 10.1038/nrc3239 22437870 PMC4856023

[119] Krummel M.F. and Allison J.P. (1995) CD28 and CTLA-4 have opposing effects on the response of T cells to stimulation. J. Exp. Med. 182, 459–465 10.1084/jem.182.2.459 7543139 PMC2192127

[120] Robert C. (2011) Ipilimumab plus dacarbazine for previously untreated metastatic melanoma. N. Engl. J. Med. 364, 2517–2526 10.1056/NEJMoa1104621 21639810

[121] Schalper K.A. (2014) *In situ* tumor PD-L1 mRNA expression is associated with increased TILs and better outcome in breast carcinomas. Clin. Cancer Res. 20, 2773–2782 10.1158/1078-0432.CCR-13-2702 24647569

[122] Nanda R. (2016) Pembrolizumab in patients with advanced triple-negative breast cancer: phase Ib KEYNOTE-012 Study. J. Clin. Oncol., 10.1200/JCO.2015.64.8931 PMC6816000 27138582

[123] Tilli T.M. (2016) Validation of a network-based strategy for the optimization of combinatorial target selection in breast cancer therapy: siRNA knockdown of network targets in MDA-MB-231 cells as an *in vitro* model for inhibition of tumor development. Oncotarget 7, 63189–63203 10.18632/oncotarget.11055 27527857 PMC5325356

[124] Dai W. (2014) Combined mTOR inhibitor rapamycin and doxorubicin-loaded cyclic octapeptide modified liposomes for targeting integrin α3 in triple-negative breast cancer. Biomaterials 35, 5347–5358 10.1016/j.biomaterials.2014.03.036 24726747

[125] Ganesan P. (2014) Triple-negative breast cancer patients treated at MD Anderson Cancer Center in phase I trials: improved outcomes with combination chemotherapy and targeted agents. Mol. Cancer Ther. 13, 3175–3184 10.1158/1535-7163.MCT-14-0358 25253784 PMC4258414

[126] Teo Z.L. (2017) Combined CDK4/6 and PI3Kα inhibition is synergistic and immunogenic in triple negative breast cancer. Cancer Res., 10.1158/0008-5472.CAN-17-2210 28947417

[127] Min A. (2015) Histone deacetylase inhibitor, suberoylanilide hydroxamic acid (SAHA), enhances anti-tumor effects of the poly (ADP-ribose) polymerase (PARP) inhibitor olaparib in triple-negative breast cancer cells. Breast Cancer Res. 17, 33 10.1186/s13058-015-0534-y 25888415 PMC4425881

[128] Borbely G. (2015) Induction of USP17 by combining BET and HDAC inhibitors in breast cancer cells. Oncotarget 6, 33623 10.18632/oncotarget.5601 26378038 PMC4741790

[129] Sekeres M.A. (2014) A randomized phase II study of azacitidine combined with lenalidomide or with vorinostat vs. azacitidine monotherapy in higher-risk myelodysplastic syndromes (MDS) and chronic myelomonocytic leukemia (CMML): North American Intergroup Study SWOG S1117. Blood 124, LBA–5 10.1200/JCO.2015.66.2510 PMC5562170 28486043

[130] Issa J.P. (2015) Results of phase 2 randomized study of low‐dose decitabine with or without valproic acid in patients with myelodysplastic syndrome and acute myelogenous leukemia. Cancer 121, 556–561 10.1002/cncr.29085 25336333 PMC4320000

[131] Prebet T. (2014) Prolonged administration of azacitidine with or without entinostat for myelodysplastic syndrome and acute myeloid leukemia with myelodysplasia-related changes: results of the US Leukemia Intergroup trial E1905. J. Clin. Oncol. 32, 1242–1248 10.1200/JCO.2013.50.3102 24663049 PMC3986386

[132] Yang A.S. and Yang B.J. (2015) The failure of epigenetic combination therapy for cancer and what it might be telling us about DNA methylation inhibitors. Epigenomics 8, 9–12, 26698294 10.2217/epi.15.94

[133] Gari H. (2016) Loss of the oncogenic phosphatase PRL-3 promotes a TNF-R1 feedback loop that mediates triple-negative breast cancer growth. Oncogenesis 5, e255 10.1038/oncsis.2016.50 27526109 PMC5007826

[134] Liang Z. (2011) Regulation of miR-19 to breast cancer chemoresistance through targeting PTEN. Pharm. Res. 28, 3091–3100 10.1007/s11095-011-0570-y 21853360

[135] Chen Y.-J. (2013) Lapatinib–induced NF-kappaB activation sensitizes triple-negative breast cancer cells to proteasome inhibitors. Breast Cancer Res. 15, R108 10.1186/bcr3575 24216290 PMC3979035

[136] Kabos P. (2011) Cytokeratin 5 positive cells represent a steroid receptor negative and therapy resistant subpopulation in luminal breast cancers. Breast Cancer Res. Treat. 128, 45–55 10.1007/s10549-010-1078-6 20665103 PMC3851293

[137] Haughian J.M. (2012) Maintenance of hormone responsiveness in luminal breast cancers by suppression of Notch. Proc. Natl. Acad. Sci. U.S.A. 109, 2742–2747 10.1073/pnas.1106509108 21969591 PMC3287001

